# From Immersion to Seizure: A Novel Case of Water Intoxication Complicating a Water Birth

**DOI:** 10.7759/cureus.84349

**Published:** 2025-05-18

**Authors:** Sarah Al-Musawi, Rukhsana Iqbal, Greeshma Rajeev

**Affiliations:** 1 Obstetrics and Gynaecology, University Hospitals of Northamptonshire, Northampton, GBR

**Keywords:** acute hyponatremia, generalized seizure, peripartum morbidity, water birth, water-electrolyte balance, water immersion, water immersion birth, water intoxication

## Abstract

Water immersion birth (WIB) is increasingly used for pain relief during labour, offering high maternal satisfaction and reduced need for epidural analgesia. However, its potential complications, particularly regarding fluid and electrolyte balance, remain underexplored. We report a case of a healthy 33-year-old woman who developed a generalized tonic-clonic seizure two hours after delivering via WIB. Her antenatal course and labour progression were clinically normal; however, she experienced retention of urine and continued fluid intake during her four hours of immersion. Post-seizure investigations revealed serum sodium measured at 124 mmol/L indicated severe hyponatremia and metabolic acidosis. Neurological imaging and EEG were unremarkable. With supportive care and correction of sodium levels, her condition stabilized. This case highlights the possibility of dilutional hyponatremia associated with prolonged WIB, especially when coupled with high fluid intake and delayed urine output. Water immersion may promote vasopressin release, impair free water clearance, and precipitate acute symptomatic hyponatremia. Although WIB is generally safe, prolonged immersion and unmonitored fluid intake may increase the risk of water intoxication. Vigilant monitoring of fluid balance is essential to prevent complications. In selected cases, serum sodium monitoring should be considered, particularly with prolonged immersion or abnormal urinary output.

## Introduction

Water immersion during labour and birth is gaining popularity in numerous nations, especially within midwifery-led care environments. Water immersion birth (WIB) is associated with a reduced need for epidural anaesthesia without affecting the mode of delivery or the rate of perineal tears [1]. In England, 6,264 out of 46,088 low- and intermediate-risk singleton term spontaneous vaginal births were delivered using WIB (representing 13.6% of births in 35 NHS Trusts) [2]. Peripartum hyponatremia is a rare condition related mainly to water intoxication that is associated with a high intake of hypotonic fluids, or it could be a result of the use of oxytocin [3]. Oxytocin acts on the V2 (vasopressin) receptor in the kidney leading to water retention; both endogenous and exogenous had affinity for these receptors explaining the possible association with dilutional hyponatremia [4]. An association between WIB and hyponatremia has not been reported previously; interestingly, prior studies investigated the effects of WIB in the initial 60 minutes or less with no additional maternal oral fluid intake which could lead to further haemodilution and the subsequent hyponatremia [5]. Water immersion has also been found to increase the rate of water retention mainly by the increased production of vasopressin as suggested by Gabrielsen et al. [6]; this water retention in addition to the oral and intravenous fluids increases the possibility of hyponatremia in WIB. The management of hyponatremia depends on correction of the cause and volume status; in more severe forms, intravenous hypertonic saline is used [7]. We present a case of a healthy woman who developed a generalized seizure (single episode) after waterbirth that was attributed to hyponatremia, highlighting the importance of careful monitoring of fluid balance during waterbirth.

## Case presentation

A 33-year-old woman, pregnant with her second child, arrived at the delivery ward at 41 weeks and three days of gestation after she had started to labour spontaneously, and she had a rupture of her membrane two hours prior to labour ward admission. She had a body mass index (BMI) of 30 and a posterior placenta which was not low, with foetal growth estimated between the 10th and 50th percentiles. Her pregnancy was otherwise uncomplicated, although she had reported a family history of epilepsy in her maternal relatives and a personal history of febrile seizures in childhood.

Upon admission, her vital signs were stable: blood pressure 139/88 mmHg, pulse 82 beats per minute, oxygen saturation 96%, and temperature 36.7°C. A vaginal examination revealed a 5-cm-dilated cervix, the baby’s head was at -2 station (mid-pelvis), and the head position was occiput posterior. Contractions were occurring three times every 10 minutes. The patient expressed a preference for a water birth, which was approved after an assessment found no contraindications.

During labour, she received oral dihydrocodeine, paracetamol, inhaled nitrous oxide (Entonox), intramuscular pethidine for pain relief, and intramuscular cyclizine. No oxytocin was given as the patient already had efficient uterine contractions. The water immersion continued for four hours in total. Despite adequate fluid intake, she did not pass urine for three hours. A urinary catheter drained 1,000 mL of urine, prompting close monitoring of fluid intake and output. At 19:38, she delivered a healthy male infant weighing 3.7 kg with Apgar scores of 8 at one minute and 10 at five and 10 minutes. Blood loss was minimal (200 mL), and the third stage of labour was actively managed.

Two hours following delivery, the patient experienced a sudden generalized tonic-clonic seizure without warning. Her husband supported her head during the 20-second episode, preventing injury. She briefly appeared cyanosed but did not have hypersalivation or lose bladder/bowel control. Post-seizure, she remained confused for three hours. Initial assessments revealed low oxygen saturation (89%), which improved to 99% with 15 L/min oxygen via a non-rebreathe mask. Blood tests showed hyponatremia (sodium 124 mmol/L), elevated lactate (13.56 mmol/L), and metabolic acidosis (pH 7.12, base deficit -14.4). Other notable results included a raised white cell count (23.2 ×10⁹/L), C-reactive protein (8 mg/L), and mild hypokalaemia (3.2 mmol/L); blood investigations just after the seizure and after one day are shown in Table 1.

**Table 1:** Serial laboratory parameters post-seizure and after 24 hours Alk. Phos: alkaline phosphatase; ALT: alanine aminotransferase; CRP: C-reactive protein; eGFR: estimated glomerular filtration rate; HB: haemoglobin; MCH: mean corpuscular haemoglobin; MCHC: mean corpuscular haemoglobin concentration; MCV: mean corpuscular volume; MPV: mean platelet volume; NRBCs: nucleated red blood cells; PCV: packed cell volume; Platelets: platelet count; RBC: red blood cells; RDW: red cell distribution width; TSH: thyroid-stimulating hormone; WBC: white blood cells.

Component	Initial Value	After One Day Value	Units	Normal Range
WBC	23.2	10.1	x10^9/L	4.0-11.0
RBC	4.74	3.72	x10^12/L	3.80-5.80
HB	129	109	g/L	115-165
PCV	0.390	0.301	L/L	0.370-0.470
MCV	82.3	80.9	fL	76.0-97.0
MCH	27.2	27.5	pg	27.0-33.0
MCHC	331	339	g/L	300-360
RDW	14.1	13.9	%	9.0-15.0
Platelets	233	175	x10^9/L	150-450
MPV	12.7	12.4	fL	7.5-11.0
Neutrophils	19.2	8.4	x10^9/L	2.0-7.5
Lymphocytes	2.3	1.2	x10^9/L	1.5-4.0
Monocytes	1.6	0.5	x10^9/L	0.2-1.0
Eosinophils	0.1	0.0	x10^9/L	0.0-0.5
Basophils	0.1	0.0	x10^9/L	0.0-0.2
NRBCs	0.0	0.0	x10^9/L	0.0-0.1
CRP	8	19	mg/L	<5
Bilirubin	16	8	µmol/L	0-21
ALT	27	22	IU/L	<33
Albumin	39	31	g/L	35-50
Alk. Phos	142	106	U/L	30-130
Total protein	67	49	g/L	60-80
Lactate	13.56	1.44	mmol/L	0.50-2.20
TSH	2.77	-	mIU/L	0.27-4.20
Amylase	88	-	IU/L	28-100
Sodium	124	134	mmol/L	133-146
Potassium	3.2	4.2	mmol/L	3.5-5.3
Urea	2.4	1.8	mmol/L	2.1-7.1
Creatinine	64	65	µmol/L	45-84
eGFR	>90.0	>90.0	ml/min/1.73m²	>90.0

Based on clinical signs, elevated inflammatory markers, and metabolic acidosis, empirical antibiotics were started following local sepsis protocols. Once blood cultures returned negative and the patient improved clinically, antibiotics were stopped.

**Figure 1:**
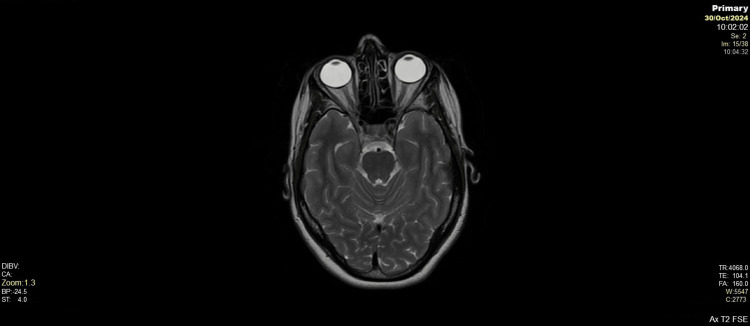
Brain axial T2-weighted MRI Axial T2-weighted MRI showing no central pontine demyelination, normal brainstem, cerebellum, temporal lobes, and basal cistern anatomy. No lesions, oedema, or structural abnormalities noted.

**Figure 2:**
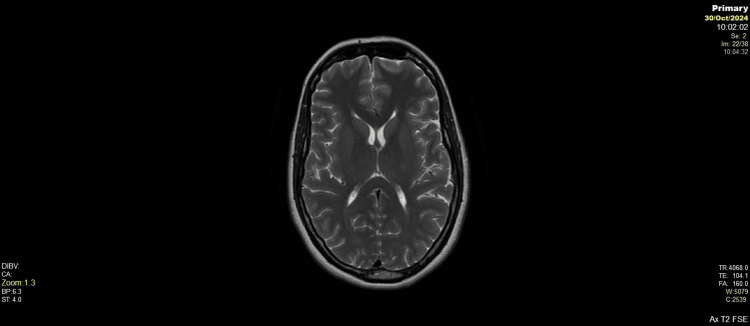
Axial T2-weighted MRI at lateral ventricles No evidence of cerebral oedema or abnormal signal intensity on T2-weighted axial images. The brain parenchyma appears normal with preserved grey-white matter differentiation. No signs of PRES or other postpartum-related intracranial abnormalities are identified.

**Figure 3:**
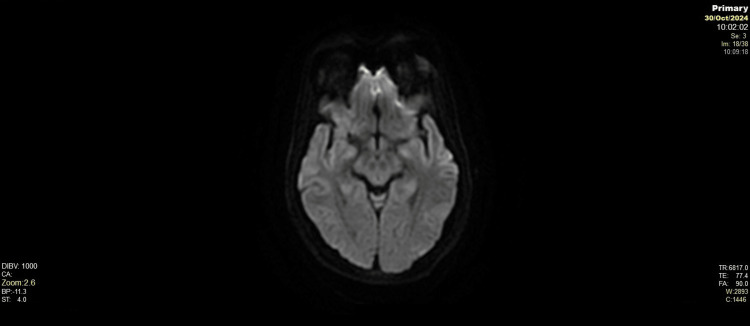
Axial diffusion-weighted imaging (DWI) No areas of restricted diffusion seen on DWI, excluding acute ischemia or cytotoxic oedema. Findings are within normal limits.

**Figure 4:**
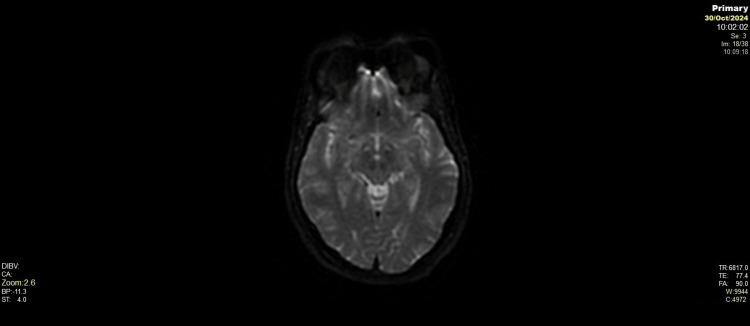
Axial apparent diffusion coefficient (ADC) map Axial ADC map confirms normal diffusion patterns across the brain with no reduction suggestive of ischemia or cytotoxic oedema. Cerebellum, brainstem, and cerebral hemispheres demonstrate homogenous diffusion properties.

The neurology team recommended correction of hyponatremia with slow intravenous fluids, a sepsis workup, and initiation of levetiracetam (1,500 mg oral loading dose followed by 500 mg twice daily). Intravenous co-amoxiclav was administered empirically. Over 24 hours, her sodium levels normalized, WBC on the second day was 10.1, and her mental clarity returned. Later on, blood culture came back negative. Follow-up brain MRI (shown in Figures 1-4) and electroencephalogram (EEG) showed no abnormalities. She was discharged in a stable condition with outpatient neurology follow-up; the antiepileptic medications were stopped after the results of EEG and MRI.

## Discussion

Hyponatremia in healthy peripartum women is an underreported condition that needs careful consideration and active investigation [3]. Water immersion birth is widely considered a safe and effective method to reduce pain and increase maternal satisfaction; it also presents physiological challenges that are not yet fully understood, particularly regarding fluid and electrolyte homeostasis [2].

Thaker [8] found that women were more prone to develop hyponatremia in the peripartum period, and it could be either a dilutional or a non-dilutional type. The dilutional hyponatremia is caused by higher intake of water, either intravenously or orally, in addition to the effect of oxytocin (both endogenous and exogenous), which acts as a potent antidiuretic hormone, leading to pronounced water retention and increasing the possibility of dilutional hyponatremia. In this case report, the patient continued to take oral fluid with initial delayed diuresis (for three hours) could be attributed to fluid retention contributing to dilutional hyponatremia.

Water immersion affects the body physiology in different mechanisms as the external pressure of the water on the body leads to redistribution of blood volume, promoting central volume expansion and the release of atrial natriuretic peptide (ANP) and suppression of renin and aldosterone, as suggested by Stadeager et al. [9]. Furthermore, immersion may also stimulate non-osmotic release of arginine vasopressin (AVP), impairing the kidneys’ ability to excrete free water and predisposing to water retention and hyponatremia [6,10]. Previous studies on water immersion mostly focused on short-term exposures to immersion and no actual consideration to ongoing fluid intake throughout labour. The current case findings highlight the importance of monitoring the fluid input in prolonged immersion as this led to additional water retention leading to a clinically significant hyponatremia.

The neurological presentation was consistent with acute symptomatic hyponatremia as a probable contributing factor to seizure, characterized by sudden onset, postictal confusion, and low serum sodium (124 mmol/L). Elevated lactate and metabolic acidosis further reflect the physiological stress associated with this seizure activity. The elevated WBC noticed initially could be explained by the period of hypoxia during the seizure attack as suggested by Huang et al. [11]. However, the possibility of concurrent infection cannot be entirely ruled out. The use of empirical antibiotics was based on initial elevated white cell count, CRP, and acidosis.

Previous studies found case of hyponatremia-induced seizure at Na level of 124 mmol/L (Halawa et al. [12]) is rare, but it is not a never incident. This highlights the importance of this case report as the patient was more prone to develop seizure even with minimal hyponatremia.

Transient urinary retention in this case may have further contributed to impaired free water clearance, compounding the dilutional effect of continued oral fluid intake during immersion. Although water intoxication-induced maternal seizure was reported previously (Walter et al. [13]), this is the only case that could connect the WIB directly to hyponatremia-induced maternal seizure. This case report highlights the importance of careful monitoring of fluid balance in WIB to avoid unpredicted electrolyte disturbance and minimizing maternal mortality in post-partum period.

Limitation of the study

There was no report of sodium level prior to the start of water immersion for the comparison. The water intake from the patient was not measured/not documented, and this could be further cause of water intoxication.

## Conclusions

Water immersion birth could be associated with an increased risk of water retention, and unregulated intake of water could lead to a cumulative effect resulting in symptomatic hyponatremia and potential maternal seizure. Careful patient selection, vigilant fluid balance monitoring, and sodium measurement should be considered, especially in cases of prolonged immersion or abnormal urinary output. This case highlights the importance of balancing the known benefits of WIB with the potential risk of water intoxication.
